# Successful reduction of intraventricular asynchrony is associated with superior response to cardiac resynchronization therapy

**DOI:** 10.1186/1476-7120-8-35

**Published:** 2010-09-01

**Authors:** Henryk Dreger, Adrian C Borges, Gert Baumann, Christoph Melzer

**Affiliations:** 1 Medizinische Klinik für Kardiologie und Angiologie, Campus Mitte, Charité - Universitätsmedizin Berlin, Germany; 2 HELIOS Klinikum Emil von Behring, Berlin, Germany

## Abstract

**Background:**

Cardiac resynchronization therapy (CRT) is generally associated with a low to moderate increase of the left ventricular ejection fraction (LVEF). In some patients, however, LVEF improves remarkably and reaches near-normal values. The aim of the present study was to further characterize these so called 'super-responders' with a special focus on the extent of intra- and interventricular asynchrony before and after device implantation compared to average responders.

**Methods:**

37 consecutive patients who underwent CRT device implantation according to current guidelines were included in the study. Patients were examined by echocardiography before, one day after and six months after device implantation. Pre-defined criterion for superior response to CRT was an LVEF increase > 15% after six months.

**Results:**

At follow-up, eight patients (21.6%) were identified as super-responders. There were no significant differences regarding age, gender, prevalence of ischemic heart disease and LVEF between average and super-responders at baseline. After six months, LVEF had significantly increased from 26.7% ± 5.7% to 33.1% ± 7.9% (*p *< 0.001) in average and from 24.0% ± 6.7% to 50.3% ± 7.4% (*p *< 0.001) in super-responders. Both groups showed a significant reduction of QRS duration as well as LV end-diastolic and -systolic volumes under CRT. At baseline, the interventricular mechanical delay (IVMD) was 53.7 ± 20.9 ms in average and 56.9 ± 22.4 ms in super-responders - representing a similar extent of interventricular asynchrony in both groups (*p *= 0.713). CRT significantly reduced the IVMD to 20.3 ± 15.7 (*p *< 0.001) in average and to 19.8 ± 15.9 ms (*p *= 0.013) in super-responders with no difference between both groups (*p *= 0.858). As a marker for intraventricular asynchrony, we assessed the longest intraventricular delay between six basal LV segments. At baseline, there was no difference between average (86.2 ± 30.5 ms) and super-responders (78.8 ± 23.6 ms, *p *= 0.528). CRT significantly reduced the longest intraventricular delay in both groups - with a significant difference between average (66.2 ± 36.2 ms) and super-responders (32.5 ± 18.3 ms, *p *= 0.022). Multivariate logistic regression analysis identified the longest intraventricular delay one day after device implantation as an independent predictor of superior response to CRT (*p *= 0.038).

**Conclusions:**

A significant reduction of the longest intraventricular delay correlates with superior response to CRT.

## Background

Conduction disorders such as left bundle branch block (LBBB) are a common finding in patients with chronic heart failure (CHF) and frequently result in intra- and/or interventricular asynchrony [1]. Since asynchrony aggravates mitral regurgitation and disturbs left ventricular (LV) contraction it consequently further promotes LV remodeling. Cardiac resynchronization therapy (CRT) aims at reverse remodeling by biventricular pacing and has been demonstrated to remarkably improve morbidity and mortality in CHF patients [2-4]. Only about 70% of all patients, however, show a significant response to CRT [2-5]. As cardiac asynchrony plays a major role in the rationale of CRT, a number of studies tried to improve patient selection for CRT by echocardiographic assessment of cardiac asynchrony. Unfortunately, the largest multi-center study to date was unable to confirm the effectiveness of twelve analyzed echocardiographic asynchrony measures to predict response to CRT [6]. Accordingly, patient selection for CRT is currently based only on LVEF, QRS duration and NYHA class [7].

Overall, the average LV ejection fraction (LVEF) increase under CRT lies in the range of 3-5% [8]. In a small subset of patients, however, CRT results in an astonishing improvement of LV function by more than 15%. Recent studies reported that duration of heart failure symptoms, presence of LBBB, left ventricular volumes and global longitudinal strain might serve as predictors for superior response to CRT [9-12]. However, data on the extent of intra- and interventricular asynchrony in super-responders compared to average responders is sparse.

## Aim

The aim of our study was to analyze the characteristics of the super-responders among our CRT patients with a special focus on intra- and interventricular asynchrony.

## Methods

### Study population

Between April 2008 and December 2009, 37 patients were included in this observational cohort study. Inclusion criteria were indication for CRT according to current guidelines (i.e., NYHA class III-IV under optimal medical therapy, LVEF ≤ 35%, LV dilation, and QRS duration ≥ 120 ms [7]), age > 18 years and ability to provide informed consent. Exclusion criteria were non-cardiac comorbidities associated with a life-expectancy < 12 months. All patients with ischemic heart disease were either tested for stress-induced ischemia by exercise tests or underwent coronary angiography before CRT.

The study conforms to local university ethics guidelines and the principles outlined in the Declaration of Helsinki.

Superior response to CRT was defined as an LVEF increase > 15% six months after implantation.

### Echocardiography

All patients were examined by echocardiography using a Vivid 7 ultrasound system (GE Medical Systems, Horton, Norway) before as well as one day and six months after device implantation. LVEF was calculated using Simpson's biplane approach [13].

### Determination of asynchrony

Asynchrony was assessed before and one day after CRT device implantation.

The right and left ventricular mechanical delays (RVMD, LVMD) were determined by measuring the interval between the beginning of the QRS complex and the opening of the pulmonary and aortic valve, respectively. Interventricular asynchrony was defined as an interventricular mechanical delay (IVMD) > 40 ms [14].

Intraventricular asynchrony was determined as described previously [15]. Briefly, the interval between the opening of the aortic valve (AVO) and the peak systolic velocity (S') was measured using tissue Doppler imaging (TDI) in six basal LV segments. Tissue synchronization imaging (TSI) was used as an internal plausibility control and confirmed correct determination of S' in patients with a reduced acoustic window. The segment with the shortest AVO-S' interval served as a reference segment as it most likely represents vital and intact myocardium. To determine asynchronous segments, we calculated the time differences between the AVO-S' intervals of the reference and the other segments. Regions were considered asynchronous when the calculated delay was above the upper limit of normal [15].

In addition, as a rough estimate of the extent of LV intraventricular asynchrony, we calculated the longest intraventricular delay, i.e., the delay between the segments with the shortest and longest AVO-S' intervals.

### Device implantation

LV leads were implanted by a transvenous approach via the coronary sinus into the lateral or posterolateral cardiac vein. All implantations were performed by two experienced board-certified cardiologists.

### AV- and VV-delay optimization

We performed AV- and VV-delay optimization in all patients according to current guidelines [7]. If asynchrony was not corrected under synchronous biventricular pacing, LV and RV pacing were programmed to the delay resulting in the shortest intraventriclar delay (maximum programmed delay: 40 ms).

AV-delay optimization was performed using the iterative method published by Cleland *et al. *[4]. In brief, we initially programmed a long AV interval (e.g., 75% of the intrinsic AV interval). The AV interval was then decreased in 20 ms steps until we observed an A-wave truncation. The AV interval was then incremented in 10 ms steps to obtain the optimal setting.

### Statistics

Data are expressed as mean ± standard deviation (SD). Statistical significance was calculated using *z*-tests, *t-*tests and paired *t*-tests when appropriate (SigmaStat 3.0, SPSS, Inc.). Multiple logistic regression analysis was performed to identify variables that are associated with superior response to CRT. An error probability of *p *< 0.05 was considered statistically significant. Diagrams depict box plots with the lower boundary indicating the 25th percentile, the upper boundary indicating the 75th percentile and a line within the box indicating the median. Whiskers above and below the box represent the 90th and 10th percentiles, respectively. Statistical outliers are marked by dots (SigmaPlot 9.0, SPSS, Inc.).

## Results

Six months after device implantation, eight patients (21.6%) were identified as super-responders. At baseline, there were no statistically significant differences regarding age, gender, prevalence of ICMP or the localization of hypokinetic segments between average and super-responders (Table 1).

**Table 1:** Baseline characteristics of the study population.

	average responders	super-responders	*p*-value
*n*	29	8	-
males, *n *(%)	24 (82.8%)	5 (62.5%)	0.455
age, years	69.7 ± 7.1	68.4 ± 7.6	0.647
ischemic cardiomyopathy, *n *(%)	13 (44.8%)	2 (25.0%)	0.545
localization of hypokinesia			
septal	0 (0%)	0 (0%)	-
lateral	1 (3.4%)	0 (0%)	0.485
anterior	0 (0%)	0 (0%)	-
inferior	3 (10.3%)	1 (12.5%)	0.639
anteroseptal	4 (13.8%)	1 (12.5%)	0.625
posterior	1 (3.4%)	0 (0%)	0.485
apical	1 (3.4%)	0 (0%)	0.485
global	20 (69.0%)	6 (75.0%)	0.915

Values are mean ± SD when appropriate.

**Figure 1:**
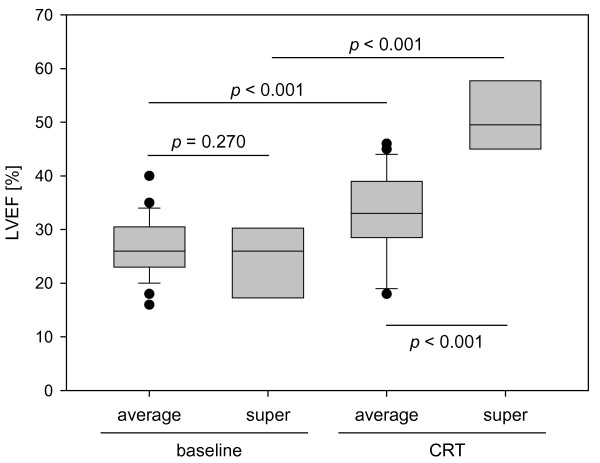
LV ejection fraction (LVEF) of average and super-responders at baseline and six months after device implantation (CRT).

At baseline, there was no statistically significant difference in the LV ejection fraction between average (26.7% ± 5.7%) and super-responders (24.0% ± 6.7%, *p *= 0.270, Figure 1). The LV end-diastolic volume (LVEDV), however, was already significantly smaller at baseline in patients who later showed superior response to CRT (*p *= 0.040, Table 2). Six months after implantation, LVEF, LVEDV, and the LV end-systolic volume (LVESV) had significantly improved compared to baseline parameters in both groups. As expected, super-responders showed significantly better LV geometry and systolic function compared to average responders.

**Table 2:** Patient characteristics at baseline and six months after device implantation.

	baseline			six months after implantation		
	average responders	super-responders	*p*-value	average responders	super-responders	*p*-value
LVEF, %	26.7 ± 5.7	24.0 ± 6.7	0.270	33.1 ± 7.9*	50.3 ± 7.4^#^	< 0.001
LVEDV, mL	199.8 ± 66.7	145.6 ± 49.4	0.040	155.0 ± 43.4*	103.4 ± 36.1^#^	0.004
LVESV, mL	145.3 ± 47.9	110.1 ± 41.6	0.067	105.7 ± 36.1*	53.0 ± 23.1^#^	< 0.001

*) *p *< 0.05 vs. average responders at baseline, ^#^) *p *< 0.05 vs. super-responders at baseline

QRS duration and prevalence of intra- and interventricular asynchrony (including the number of asynchronous LV segments and the longest intraventricular delay) did not differ significantly between both groups at baseline (Table 3). Under CRT, QRS duration significantly decreased in both groups compared to baseline but again showed no difference between average and super-responders. The right ventricular mechanical delay (RVMD) increased significantly in average and non-significantly in super-responders. Similarly, there was a significant reduction of the left ventricular mechanical delay (LVMD) in average and a non-significant LVMD decrease in super-responders. Taken together, this resulted in a significant reduction of the interventricular delay in both average and super-responders. Consequently, the prevalence of interventricular asynchrony decreased significantly in both groups. Interestingly, there were no differences in RVMD, LVMD, IVMD, and the prevalence of interventricular asynchrony under CRT between both groups.

**Table 3:** QRS duration and cardiac asynchrony parameters before and one day after device implantation.

	before implantation			after implantation		
	average responders	super-responders	*p*-value	average responders	super-responders	*p*-value
QRS, ms	153.8 ± 17.6	150.0 ± 15.1	0.583	123.8 ± 12.4*	113.8 ± 11.9^#^	0.086
RVMD, ms	88.8 ± 24.7	85.0 ± 26.2	0.708	111.6 ± 27.4*	105.5 ± 21.1	0.565
LVMD, ms	142.4 ± 25.3	141.9 ± 19.8	0.953	122.2 ± 32.2*	123.8 ± 19.7	0.567
IVMD, ms	53.7 ± 20.9	56.9 ± 22.4	0.713	20.3 ± 15.7*	19.8 ± 15.9^#^	0.858
interventricular asynchrony, *n *(%)	19 (65.5%)	6 (75.0%)	0.936	2 (6.9%)*	1 (12.5%)^#^	0.828
intraventricular asynchrony, *n *(%)	28 (96.6%)	8 (100.0%)	0.485	23 (79.3%)	4 (50.0%)	0.229
longest intraventricular delay, ms	86.2 ± 30.5	78.8 ± 23.6	0.528	66.2 ± 36.2*	32.5 ± 18.3^#^	0.022
asynchronous segments, *n *(%)	2.3 ± 1.1	2.0 ± 0.8	0.518	2.0 ± 1.2	1.1 ± 1.6	0.150

*) *p *< 0.05 vs. average responders at baseline, ^#^) *p *< 0.05 vs. super-responders at baseline.

Values are mean ± SD when appropriate.

**Figure 2:**
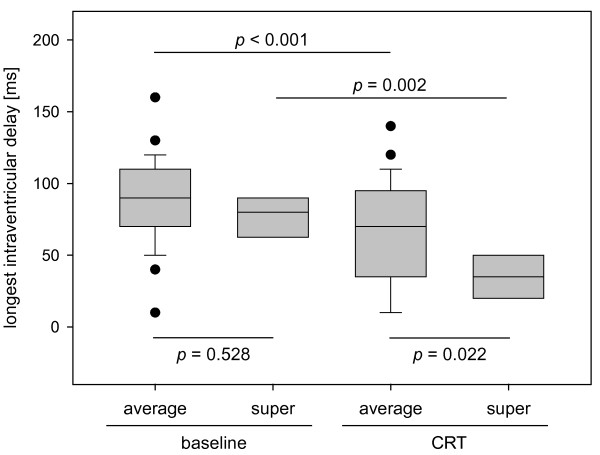
The longest intraventricular delay in average and super-responders at baseline and one day after device implantation (CRT).

**Figure 3:**
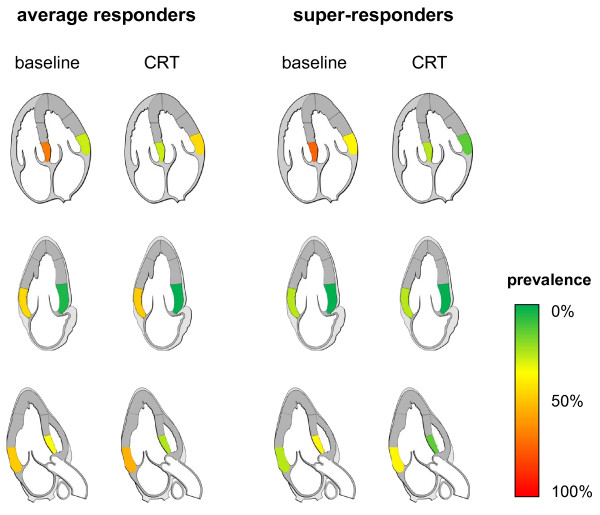
**Prevalence of intraventricular asynchrony in six basal segments in average and super-responders before and one day after device implantation (CRT). **Upper panel: septal and lateral segments in the four chamber view. Middle panel: anterior and inferior segments in the two chamber view. Lower Panel: anteroseptal and posterior segments in the three chamber view. Illustrations were originally created by Patrick J. Lynch and C. Carl Jaffe, MD and modified with permission under the Creative Commons Attribution 2.5 License 2006.

Intraventricular asynchrony (as defined by at least one asynchronous LV segment) was detected in all but one patient at baseline and there was no difference between average and super-responders regarding the longest intraventricular delay (86.2 ± 30.5 ms vs. 78.8 ± 23.6 ms, *p *= 0.528). After implantation, the prevalence of intraventricular asynchrony only non-significantly decreased in both groups. In both average and super-responders, however, the longest intraventricular delay decreased significantly to 66.2 ± 36.2 ms (*p *< 0.001) and 32.5 ± 18.3 ms (*p *= 0.002), respectively - with a significant difference between both groups (*p *= 0.022, Figure 2). There were no statistically significant differences regarding the localization of asynchronous segments between average and super-responders - neither before nor after device implantation. It is noteworthy, though, that average responders had a more evenly distributed pattern of asynchronous segments while super-responders had primarily septal asynchrony at baseline (Figure 3).

Multiple logistic regression analysis based on gender, age, etiology of heart failure, QRS duration, LVEDV, IVMD, and the longest intraventricular delay at baseline identified LVEDV as the only significant baseline predictor of superior response to CRT (*p *= 0.031). Interestingly, multiple logistic regression analysis using gender, age, etiology of heart failure as well as postoperative QRS duration, IVMD, and the longest intraventricular delay revealed that the longest intraventricular delay one day after device implantation might serve as a predictor for superior response to CRT (*p *= 0.038).

## Discussion

Cardiac resynchronization therapy represents a powerful tool to improve morbidity and mortality of CHF patients who remain symptomatic under optimal medical therapy and meet current CRT inclusion criteria [7]. Unfortunately, only about 70% of all CRT patients show a positive response after implantation which - given its high costs and invasive nature with possible severe side effects - is disappointingly low. As correction of cardiac asynchrony is a major rationale of CRT, a number of studies tried to improve patient selection by adding asynchrony assessment to CRT inclusion criteria. Unfortunately, however, the PROSPECT trial failed to confirm the effectiveness of several established echocardiographic asynchrony measures to predict response to CRT [6].

The aim of the present study was to further identify characteristics of super-responders to CRT with a special focus on inter- and intraventricular asynchrony.

Superior response to CRT as defined by an LVEF increase > 15% six months after implantation was found in 21.6% of our patients. This is consistent with previous studies on super-responders which reported a prevalence of 9.7-37.8% [9-12]. In these reports, super-responders were generally found to be more often female and to suffer less often from ischemic heart disease [10-12]. Here, we also observed a similar, but non-significant trend in our patients (Table 1). Significant differences between super- and average responders were documented regarding the LV end-diastolic volume at baseline as well as six months after implantation (Table 2). This is in agreement with earlier studies that also reported lower baseline LV volumes in super-responders [9-11]. In our opinion, however, these results are unlikely to have a large impact on patient selection in the clinical routine since they only give us information on who is likely to benefit the most from CRT. As these results do not imply that other patients will not benefit from CRT at all, it would be false to deny CRT to patients who do not share 'super-responder characteristics' (e.g., male CHF patients with ischemic cardiomyopathy and major LV dilation).

Analysis of interventricular asynchrony showed no differences between average and super-responders at baseline and after device implantation. In both groups, biventricular pacing resulted in a significant reduction of the interventricular mechanical delay (IVMD) and, consequently, a correction of interventricular asynchrony in almost all patients (Table 3). At baseline, the overwhelming majority of patients had signs of intraventricular asynchrony (Table 3). After implantation, intraventricular asynchrony was still detectable in 79.3% of the average and 50.0% of the super-responders representing a non-significant reduction in both groups (*p *= 0.229). Interestingly, however, the longest intraventricular delay was significantly reduced by 20.0 ± 25.4 ms in average and by 46.3 ± 26.7 ms in super-responders. This is consistent with a study by Antonio *et al. *who reported a reduction of the longest intraventricular delay under CRT by 25 ms in average and by 70 ms in super-responders [9]. With both groups having similar baseline values, the longest intraventricular delay was significantly shorter in our super-responders (32.5 ± 18.3 ms) compared to average responders (66.2 ± 36.2 ms) under CRT (*p *= 0.022). Furthermore, super-responders showed a trend for less asynchronous LV segments (1.1 ± 1.6) than average responders (2.0 ± 1.2) at follow-up. Not the least due to the low number of patients, there were no significant differences regarding the location of intraventricular asynchrony between both groups (Figure 3).

In the PROSPECT trial, all tested asynchrony measures failed to predict response to CRT. To some degree, this can be explained by the shortcomings of current indices. In general, however, echocardiography is *per se *not able to foresee the potential of the left ventricle to undergo reverse remodeling. Accordingly, even near-perfect assessment of asynchrony would not be able to predict response to CRT. In the present study, superior response to CRT did not correlate with the extent of cardiac asynchrony at baseline. This finding might help to understand the disappointing results of the PROSPECT trial. Importantly, however, a greater reduction of the longest intraventricular delay was associated with superior response to CRT in our study. This suggests that cardiac asynchrony is indeed a prerequisite of successful CRT and its correction should remain in our focus. In our opinion, asynchrony measurements should not serve as predictors of response to CRT. Instead, asynchrony quantification should be performed to direct VV delay optimization - aimed at minimization of the intraventricular delay.

### Limitations

Our study is clearly limited by the low number of patients. Furthermore, all data were collected at a single center. We also did not assess global contractile reserve which has recently been shown to correlate with response to CRT [16].

## Conclusions

Our results confirm once more that LV geometry at baseline is an important factor for positive response to CRT. In addition, our data suggest that not the initial extent of cardiac asynchrony but rather a successful reduction of the intraventricular delay correlates with superior response to CRT. Pending confirmation of our results by further studies, we speculate that routine assessment of intraventricular asynchrony before and after device implantation combined with a VV-delay optimization aimed at reduction of the longest intraventricular delay might help to improve response to CRT.

## List of abbreviations

AVO: aortic valve opening; CHF: chronic heart failure; CRT: cardiac resynchronization therapy; IVMD: interventricular mechanical delay; LBBB: left bundle branch block; LV: left ventricular; LVEF: left ventricular ejection fraction; LVEDV: left ventricular end-diastolic volume; LVESV: left ventricular end-systolic volume; LVMD: left ventricular mechanical delay; RVMD: right ventricular mechanical delay; S': peak systolic velocity; SD: standard deviation; TDI: tissue Doppler imaging; TSI: tissue synchronization imaging.

## Competing interests

The authors declare that they have no competing interests.

## Authors' contributions

CM established the registry, performed device implantations and examined patients before and after surgery. HD analyzed the data, performed the statistical analysis and wrote the manuscript. ACB and GB interpreted the data and critically revised the manuscript.

All authors have read and approved the final manuscript.
